# Incidence and Characteristics of Hospital‐Acquired Pressure Injuries in Acute Palliative Care Patients: A Four‐Year Analysis

**DOI:** 10.1111/jocn.17829

**Published:** 2025-05-19

**Authors:** Saroeun Ven, Michael Steele, Adam Burston, Paul Fulbrook, Josephine Lovegrove, Sandra Miles, Susan Prince

**Affiliations:** ^1^ Faculty of Health Sciences, School of Nursing, Midwifery & Paramedicine Australian Catholic University Brisbane Queensland Australia; ^2^ Nursing Research and Practice Development Centre The Prince Charles Hospital Brisbane Queensland Australia; ^3^ National Health and Medical Research Council Centre of Research Excellence in Wiser Wound Care Griffith University Gold Coast Queensland Australia; ^4^ School of Nursing, Midwifery and Social Work The University of Queensland and UQ Centre for Clinical Research Herston Queensland Australia; ^5^ Herston Infectious Diseases Institute, Metro North Health Brisbane Queensland Australia; ^6^ Palliative Care Unit The Prince Charles Hospital Brisbane Queensland Australia

**Keywords:** acute patients, palliative care, pressure injury

## Abstract

**Aim:**

To describe the cumulative incidence and characteristics of hospital‐acquired pressure injury in acute palliative patients.

**Design:**

Secondary data analysis of hospital‐acquired pressure injuries during 2019–2022.

**Methods:**

The setting was a palliative care unit at a tertiary hospital in Queensland, Australia, including adult (≥ 18 years) acute‐phase palliative inpatients. Retrospective data from four databases were used to identify and analyse hospital‐acquired pressure injury cases from 2019 to 2022. Clinical characteristics of patients with and without hospital‐acquired pressure injury were compared.

**Results:**

The incidence of hospital‐acquired pressure injury in acute palliative care patients was 3.9% over the 4 years. These patients were predominantly male, with an average age of 74 years, with 66 of 78 cases developing in the deteriorating palliative care phase. Using the Waterlow Score, 51.3% of patients were assessed as at very high risk of pressure injury. Ninety‐five hospital‐acquired pressure injuries were reported in 78 patients; 16.8% were medical device‐related, 40% were Stage 1 injuries, and the most common injury sites were the sacrum, heels and genitals. Patients with hospital‐acquired pressure injury had significantly higher (worse) scores on both the palliative care Resource Utilisation Group‐Activities of Daily Living and Problem Severity Scores. Regression analysis identified a high Problem Severity Score on admission as a significant predictor for hospital‐acquired pressure injury development.

**Conclusion:**

The incidence of hospital‐acquired pressure injury in acute palliative patients is lower than in previous studies. However, many injuries occurred in those in the deteriorating phase, with higher scores for severity of symptoms. These findings suggest that acute palliative patients do require nursing care for pressure injury prevention, as well as for symptom management and activities‐of‐daily‐living. Overall, this research contributes to a deeper understanding of pressure injury incidence and characteristics for acute palliative care patients. Future research should focus on population‐specific pressure injury risk assessment to explore risk factors in greater detail.

**Implications for the Profession and/or Patient Care:**

Current pressure injury risk assessment tools, like the Waterlow Score, may not provide the comprehensive evaluation needed for the acute palliative care cohort. To better address the unique needs of this cohort, it may be necessary to refine existing tools or develop new instruments that integrate palliative‐specific assessments, such as the Resource Utilisation Group‐Activities‐of‐Daily‐Living (RUG‐ADL) and Problem (symptom) Severity Score (PSS). These adaptations could help improve pressure injury prevention care planning and enhance outcomes for patients in this setting.

**Impact:**

This study separated acute palliative care patients from those at end‐of‐life and found a 3.9% cumulative incidence of pressure injuries. There were no significant differences in age, gender, or cancer diagnosis between patients with and without injuries. Patients without injuries were more likely to be in the deteriorating phase, while those with injuries had higher (worse) RUG‐ADL scores. Regression analysis showed that each one‐point increase in the PSS (symptom severity) made patients 1.2 times more likely to develop a pressure injury. The findings suggest that combining a validated risk assessment tool with the RUG‐ADL and PSS tools could provide a more accurate risk assessment for hospitalised acute palliative care patients.

**Reporting Method:**

STROBE reporting guideline.

**Patient or Public Contribution:**

No patient or public contribution.

**Abbreviations**
AKPS: Australia‐modified Karnofsky Performance Status
EoL: end‐of‐life
PCOC: Palliative Care Outcome Collaboration
PC‐Phase: Palliative‐Care Phase
PSS: Problem Severity Score
QHERS: Queensland Health Enterprise Reporting Service
QuEST: Quality Effectiveness Support Team
RUG‐ADL: Resource Utilisation Group—Activities of Daily Living
SAS: Symptom Assessment Scale

**Summary**

- What does this paper contribute to the wider global community?
  ○ This paper addresses a significant gap in research regarding pressure injury incidence in acute palliative care settings. It highlights the under‐researched nature of pressure injury risk assessment in this specific cohort, emphasising the need for more robust studies to understand incidence and risk factors. By proposing an acute palliative‐specific assessment tool for pressure injury prevention, the paper encourages a more nuanced, evidence‐based approach to nursing practice in this field.
  ○ Introducing such an assessment tool could improve clinical outcomes by providing a structured framework to manage pressure injury risks in acute palliative care, an area under‐researched compared to end‐of‐life care. This paper fills a crucial gap in the literature, broadening the scope of pressure injury research. Future studies could refine the tool by incorporating elements from the RUG‐ADL and PSS for a more tailored approach.
  ○ Ultimately, this paper promotes evidence‐based practices that can lead to better patient care, improved nursing knowledge and potentially reduce pressure injury occurrences in acute palliative settings.

## 1 Introduction

Pressure injuries are described as ‘localised damage to the skin and/or underlying soft tissue usually over a bony prominence’ (EPUAP et al. 2019, 16), which occur as a result of prolonged pressure or pressure in combination with shear (EPUAP et al. 2019). Globally, pressure injury remains problematic in all care settings. In Australia, hospital‐acquired pressure injury leads to poorer health outcomes including pain, reduced quality of life (QoL) and, in severe cases, increased mortality for patients (Burston et al. 2023; Song et al. 2019), while for healthcare facilities, it increases costs as a result of longer hospital stay and pressure injury treatment (Nghiem et al. 2022). For this reason, hospital‐acquired pressure injury is regarded as an adverse event associated with hospitalisation, with severe injuries classified officially as *Hospital Acquired Complications* (Australian Commission on Safety and Quality in Health Care [ACSQHC] 2021). Thus, pressure injury occurrence (incidence and prevalence) as well as an individual's risk factors and level of risk are routinely assessed in hospital settings across all patient cohorts.

## 2 Background

Originating from the hospice movement (Ferris et al. 2019), palliative care was traditionally associated with end‐of‐life (EoL) care, predominantly for patients with terminal cancer. However, in the 1970s, the scope of palliative care expanded beyond institutionalised hospice care to include all individuals living with life‐limiting illnesses, such as those with chronic or non‐cancer diagnoses (Hudson et al. 2021). Today, while EoL care remains a core component of palliative care, its scope is broader, aiming to alleviate suffering and enhance QoL for individuals at any stage of a life‐limiting illness, not just at the end of life (World Health Organisation [WHO] 2023).

In 1993, a draft case‐mix classification for palliative care was proposed, identifying five distinct phases: stable, acute, deteriorating, terminal and post‐death bereavement (Masso et al. 2015). This classification was refined in 1994, with terminology adjusted to stable, unstable, deteriorating, terminal and bereavement (Masso et al. 2015). These phases are now embedded into standard assessment tools widely used in the Palliative Care Outcome Collaboration (PCOC, University of Wollongong 2020), which offers a framework for determining appropriate care at each stage. These phases are defined as follows:

1. *Stable phase*: Symptoms are well managed under an established care plan.
2. *Unstable phase*: A sudden change in condition necessitates urgent adjustments to the care plan.
3. *Deteriorating phase*: Symptoms progressively worsen, requiring periodic review of the care plan to address evolving needs.
4. *Terminal phase*: Death is anticipated within days (Masso et al. 2015).

For this study, an ‘acute palliative care patient’ refers to an individual in any of the first three phases (stable, unstable, or deteriorating) admitted to a palliative care unit, typically for active treatment or symptom management to improve QoL.

Pressure injuries are a significant concern in palliative care, with incidence rates ranging from 2.5% to 35% across various settings, including hospital inpatient Palliative Care Units, home care and hospices (Artico et al. 2018; Ferris et al. 2019). A Polish study (Sternal et al. 2017) found 25.5% of 329 hospitalised palliative care patients were admitted with existing pressure injuries, and 11.9% developed new ones during their stay. A review of studies undertaken in the United Kingdom, United States and Canada (Kaltenthaler et al. 2001) identified a 1996 study (Hatcliffe and Dawe 1996) reporting that the highest prevalence of pressure injuries (37%) was in the Palliative Care Unit compared to other healthcare settings, such as hospitals, nursing homes and community care.

Most studies on pressure injuries in palliative care have focused on hospice settings, with fewer addressing hospital‐acquired pressure injuries among acute palliative care patients (Artico et al. 2018; Ferris et al. 2019; Nenna 2011). In the United Kingdom, a 2‐year audit in a specialist palliative care unit found that 12% of 542 patients developed a pressure injury during their stay, with 95.3% of patients assessed as having a high‐ or very high‐risk of pressure injury development (Galvin 2002). Most research has focused on patients in the terminal phase of illness (Ferris et al. 2019), with limited attention given to those in the acute phases. Additionally, the terms ‘palliative care’ and ‘palliative patient’ are inconsistently defined across studies (Ferris et al. 2019; Hatcliffe and Dawe 1996). Given the pathophysiological differences in pressure injury development for palliative patients at EoL (terminal phase), such as reduced skin and soft tissue blood perfusion associated with skin failure (Ferris et al. 2019), it is important to examine acute palliative care and EoL cohorts separately. Therefore, this study focused exclusively on acute palliative care patients.

Hospitals routinely use a tool to assess each patient and identify their individual pressure injury risk factors to determine their level of risk for developing pressure injuries. The Waterlow score (Waterlow 2005) is commonly used in Queensland hospital settings, including the study setting, for assessing patients in acute and palliative care, though it is not designed for the specific palliative care cohort. The risk‐level score assists nurses to determine the most appropriate pressure injury prevention strategies to implement to mitigate that risk (Fulbrook et al. 2024). Risk factors and levels are important characteristics to study when describing pressure injury incidence, leading to the study aim.

## 3 The Study

### 3.1 Aim

To describe the incidence, risk factors, risk level and other characteristics of hospital‐acquired pressure injury in adult acute palliative care patients in a hospital setting from 2019 to 2022.

### 3.2 Objectives

1. Identify the cumulative incidence of hospital‐acquired pressure injuries in adult acute palliative care patients over a 4‐year period from 2019 to 2022.
2. Identify the characteristics of palliative care patients who develop hospital‐acquired pressure injuries versus those who did not.
3. Identify the predictors of hospital‐acquired pressure injuries for this cohort.
4. Examine the characteristics of the identified pressure injuries in this cohort.

## 4 Methods

### 4.1 Design

Using hospital databases and patient medical records, a secondary data analysis was undertaken of reported hospital‐acquired pressure injury incidents. A comparison was made of the clinical characteristics of palliative care patients with hospital‐acquired pressure injuries and those without in the acute phases (stable, unstable, deteriorating) of palliative care. The study is reported following Strengthening the Reporting of Observational Studies in Epidemiology (STROBE) guidelines (Von Elm et al. 2007) (Data S1).

### 4.2 Study Setting and Sample

The study setting was a tertiary hospital in southeast Queensland, Australia. The facility has a 16‐bed palliative care unit, which provides care to patients with a broad case mix of malignant and non‐malignant diseases and admits people in all phases of their illness.

### 4.3 Inclusion Criteria

The sample included all adult (aged ≥ 18 years) acute‐phase palliative care inpatients admitted to the palliative care unit between 2019 and 2022.

### 4.4 Data Sources

The hospital's Clinical Health Information Service provided a list of patients admitted under the palliative care treatment team. Data on all admitted inpatient palliative care patients, including Waterlow scores, were extracted, including a subset for hospital‐acquired pressure injuries, covering the period from January 2019 to December 2022. Missing data were supplemented through the review of paper‐based patient medical records.

The study hospital utilises a modified version of the Waterlow Score (Waterlow 2005) to assess each patient and identify their individual pressure injury risk factors to determine their level of risk for developing pressure injuries. This risk assessment tool evaluates factors such as build/weight for height, continence, skin type, mobility, sex, age and appetite. It also includes a special risk category for tissue malnutrition, neurological deficit, major surgery/trauma and medication. These factors are then scored and summed to categorise the pressure injury risk level: < 10 = not at risk; ≥ 10–14 = at risk; ≥ 15–19 = high risk; ≥ 20 = very high risk (Waterlow 2005). The modified Waterlow tool at the study hospital incorporates a Malnutrition Screening Tool (MST; Ferguson et al. 1999) with questions about recent weight loss and appetite, with scores of 2 or more requiring dietitian referral.

### 4.5 Data Were Obtained From Four Data Sources

#### 4.5.1 Riskman

Data on patients having hospital‐acquired pressure injuries (both all‐stage hospital‐acquired and severe‐stage community‐acquired) were documented using an electronic incident reporting system (RiskMan) during the data collection period. The RiskMan system records all adverse incidents experienced by patients during hospitalisation.

#### 4.5.2 Quality Effectiveness Support Team

The Quality Effectiveness Support Team (QuEST) conducted audits of all hospital‐acquired pressure injuries to review and validate their presence and stage. In cases where QuEST could not validate a pressure injury, the RiskMan data were used.

#### 4.5.3 Queensland Health Enterprise Reporting Service

To assess the completeness of incident reporting for acute palliative care patients, data from the QuEST database and the RiskMan incident reporting system were compared with data extracted from the Queensland Health Enterprise Reporting Service (QHERS) database. QHERS performs a separate auditing process for all hospital patients based on chart review. Trained clinical coders review and code medical records after patient discharge, identifying severe hospital‐acquired pressure injuries (stage 3, stage 4, unstageable, suspected deep tissue injury).

#### 4.5.4 Palliative Care Outcome Collaboration

The PCOC database was utilised to extract data for all palliative care patients to distinguish between those in acute palliative care phases and those in the terminal phase. In Australia, the PCOC framework is integrated into clinical practice, incorporating assessments to identify and address inpatient needs and to benchmark service delivery across public, private and community sectors. The study hospital collects PCOC data, maintaining a database of all palliative care patients, including their care phase (stable, unstable, deteriorating) at the time of admission. PCOC data include patient demographics (e.g., Unique Hospital Number [URN], gender, age) and primary diagnosis.

Furthermore, as part of PCOC data, several tools are used to assess illness and symptom severity at admission. Admission scores from these tools were extracted from the hospital's PCOC database (Data S2).

##### 4.5.4.1 Australia‐Modified Karnofsky Performance Status

The Australia‐modified Karnofsky Performance Status (AKPS), completed by clinicians, measures a patient's performance in activity, work and self‐care on a scale from 10 to 100, with 100 indicating full independence and 10 indicating coma or minimal responsiveness (Abernethy et al. 2005).

#### 4.5.5 Problem Severity Score

The Problem Severity Score (PSS) assesses symptom severity across four domains: pain, psychological/spiritual, other symptoms and family/carer, with ratings from 0 (absent) to 3 (severe) for screening, management and care coordination (Eagar et al. 2004).

#### 4.5.6 Resource Utilisation Group—Activities of Daily Living

The Resource Utilisation Group—Activities of Daily Living (RUG‐ADL) is a four‐item scale assessing functional status in bed mobility, toileting, transfers and eating. It identifies needed assistance and resources, focusing on what the patient does, not how they perform (Eagar et al. 2004). Each item is scored based on the level of assistance required, with higher scores indicating greater dependency. The individual scores are summed to produce a total RUG‐ADL score ranging from 4 to 16, where scores of 4–8 reflect low care needs, 9–12 indicate moderate care needs and 13–16 represent high care needs.

#### 4.5.7 Symptom Assessment Scale

The Symptom Assessment Scale (SAS) is a tool where patients or a proxy rate distress from common symptoms—pain, fatigue, nausea, breathlessness, anxiety, depression and insomnia—on a 0–10 scale, 0 being distress‐free and 10 being severe distress (Kristjanson et al. 1999).

### 4.6 Data Analysis

Data from all sources were merged into a single Microsoft Excel database, where they were checked and cleaned before being imported into IBM SPSS (version 28) for analysis. All patients who developed a pressure injury while still in an acute phase were identified as a subset. Patient demographics were analysed using descriptive statistics, and the sample characteristics are described using means (*M*) with standard deviation (SD) or medians (Md) with interquartile range (IQR). The Mann–Whitney *U* Test was used to compare differences between groups, and the Chi‐squared test was used to explore the relationship between patients with hospital‐acquired pressure injury and those without.

Cumulative incidence was used as a reliable measure of the proportion of the studied population that developed a new pressure injury across the time period. Hospital‐acquired pressure injury incidence was calculated as: number of cases [numerator]/admission episodes [denominator] × 100% (Baharestani et al. 2009), where the number of patient cases that developed a pressure injury between 2019 and 2022 was divided by the total number of patient cases (admission episodes) over the same period.

Direct logistic regression (forced entry) was used to determine predictive factors (Hosmer et al. 2013) for hospital‐acquired pressure injury against a set of selected predictor variables, including gender, deteriorating phase, AKPS score, PSS score and RUG‐ADL score. Data for these variables were available for both acute palliative care patient groups: those with hospital‐acquired pressure injuries and those without; hence their selection. All predictor variables were entered into the model in a single block to evaluate their predictive ability while controlling for the influence of the other variables (Pallant 2020). Statistical significance was set at *p* < 0.05 for all analyses.

## 5 Ethical Considerations

Ethical approval was received from the hospital's Human Research Ethics Committee (reference: HREC/2023/MNHB/90898) and registered with the university Human Research Ethics Committee (reference: ACU 2023‐3195R). Relevant data custodians approved access to hospital databases and patient records via Public Health Act approval (reference: PHA 90898). Individual patient consent was not required as a waiver of consent was approved.

## 6 Results

### 6.1 Objective 1

During the study period, there were 3045 admissions (patient cases) to the palliative care unit. Following the exclusion of EoL patients (*n* = 539) and non‐palliative care medical outliers (*n* = 530), there were 1976 acute‐phase admissions. Of these, 95 patient cases were identified initially with at least one hospital‐acquired pressure injury. However, 15 cases were excluded as the pressure injury was acquired prior to transferring into the palliative care ward, and a further two cases were excluded as they were acquired in another hospital prior to admission. Thus, with 78 patient cases, the cumulative incidence of hospital‐acquired pressure injury in acute‐phase palliative care patients was calculated as 3.9%. In total, 95 pressure injuries were identified.

### 6.2 Objective 2

#### 6.2.1 Characteristics of the Sample

The overall sample for palliative care patients in the three acute phases (stable, unstable, deteriorating) was *n* = 1976. The mean age of the total sample (*n* = 1976) was 76.2 years (SD 10.6, range 25–95) and most were male (57.4%, *n* = 1135). A cancer diagnosis was present in 43.8% (*n* = 865) of cases. The majority (68.7%, *n* = 1357) were categorised as being in a deteriorating phase. There were significantly higher (worse) admission RUG‐ADL and PSS scores in patients who developed pressure injuries compared to those who did not (see Table 1).

**TABLE 1:** Sample characteristics (*n* = 1976).

Characteristics	Hospital‐acquired pressure injury		Significance
	Yes (*n* = 78)	No (*n* = 1898)	*p*
Age in years, mean (SD)	75.0 (11.7)	76.3 (10.6)	0.281^a^
Gender, % (*n*)			
Male	65.4 (51)	57.1 (1084)	0.148^a^
Female	34.6 (27)	42.9 (814)	
Cancer diagnosis, % (*n*)	44.9 (35)	43.7 (830)	0.842^a^
Palliative care phase			
1: Stable	3 (3.8)	132 (7.0)	0.431^a^
2: Unstable	17 (21.8)	467 (24.6)	
3: Deteriorating	58 (74.4)	1299 (68.4)	
AKPS index, median (IQR)	30 (20–40)	30 (30–50)	0.096^b^
RUG‐ADL, median (IQR)			
Bed mobility (score 1, 3, 4, 5)	4 (3–5), 1–5	3 (1–5), 1–5	0.027^b^
Toileting (score 1, 3, 4, 5)	4 (3–5), 1–5	3 (3–5), 1–5	0.040^b^
Transfer (score 1, 3, 4, 5)	4 (3–5), 1–5	3 (3–5), 1–5	0.031^b^
Eating (score 1, 2, 3)	2 (1–2), 1–3	1 (1–2), 1–3	0.014^b^
Total score (range 4–18)	14 (10–17), 4–18	11 (8–16), 4–18	0.017^b^
RUG‐ADL at pressure injury risk (score ≥ 15), % (*n*)	42.3 (33)	34.2 (650)	0.142^a^
PSS, median (IQR), range (item score range 0–3)			
Pain	1 (0–1), 0–2	1 (0–1), 0–3	0.668^b^
Other symptoms	1 (1–1.25), 0–3	1 (0–1), 0–3	0.010^b^
Psychological/spiritual	1 (1–1), 0–3	1 (0–1), 0–3	0.002^b^
Family	1 (1–1), 0–3	1 (0–1), 0–3	0.003^b^
Total score	4 (3–5), 0–11	3 (2–4), 0–12	0.003^b^
SAS, median (IQR), range (item score range 0–10)			
Insomnia	0 (0–1.25), 0–8	0 (0–1), 0–10	0.582^b^
Appetite	0 (0–3), 0–8	0 (0–2), 0–10	0.104^b^
Nausea	0 (0–0), 0–7	0 (0–0), 0–10	0.243^b^
Bowels	0 (0–3), 0–9	0 (0–2), 0–10	0.049^b^
Breathing	0 (0–3), 0–9	0 (0–3), 0–10	0.814^b^
Fatigue	2 (0–5), 0–8	2 (0–4), 0–10	0.245^b^
Pain	2 (0–5), 0–9	2 (0–5), 0–10	0.824^b^
Total score	9.5 (4–18), 0–49	8 (4–15), 0–53	0.390^b^

Abbreviations: AKPS, Australia‐modified Karnofsky Performance Status; PSS, Problem Severity Score; RUG‐ADL, Resource Utilisation Group—Activities of Daily Living; SAS, Symptom Assessment Scale.

^a^ Chi‐squared test.

^b^ Mann–Whitney *U* test.

#### 6.2.2 Characteristics of Patients With Pressure Injury

The mean age of acute‐phase palliative care patients (cases) with hospital‐acquired pressure injury was 74.0 years (SD 11.7, range 32–9). For both genders there were high proportions aged ≥ 65 years (male = 88.2%, *n* = 45; female = 74.1%, *n* = 20). Almost half of the cases (*n* = 36; 46.2%) were intra‐hospital ward transfers. The mean Waterlow pressure injury risk score on admission was 20.2 (SD 6.1) with most categorised as very high risk (51.3%, *n* = 40) and none categorised as not at risk, whereas the RUG‐ADL categorised 57.7% (*n* = 45) of patients as not at risk (score < 15). Based on the MST score (range 1–5), which is a component of the Waterlow Score used in the study setting, most patients were at high risk (42.3%, *n* = 33) of malnutrition (see Table 1).

At the time of admission, most patients (84.6%, *n* = 66) were classified as being in the deteriorating phase (see Table 2). Eleven admission diagnoses were reported, of which the most common was the *International Classification of Disease 11: Neoplasms* (44.9%, *n* = 35). Patients were admitted with up to six comorbid conditions (Md 3, IQR 2–4); ten different comorbidities were reported in 75 patients, of which the most common was diabetes (57.7%, *n* = 45). All patients were receiving multiple medications (Md 12, IQR 6–13, range 2–16), with 19 different classes of drug prescribed. The median palliative care unit length of stay was 14 days (IQR 7–27, range 2–72) and the majority deteriorated further and died whilst in the unit (84.6%, *n* = 66).

**TABLE 2:** Characteristics of patients with hospital‐acquired pressure injury (*n* = 78).

Previous history of pressure injury, % (*n*)	34.6 (27)
Palliative care phase at time of pressure injury, *n* (%)	
1. Stable	3 (3.8)
2. Unstable	9 (11.5)
3. Deteriorating	66 (84.6)
Waterlow admission score, mean (SD)	20.2 (6.1)
Waterlow admission risk category, % (*n*)	
Not at risk	0
At risk	24.4 (19)
High risk	24.4 (19)
Very high risk	51.3 (40)
RUG‐ADL at risk of pressure injury (score ≥ 15), % (*n*)	
Not at risk	37.2 (29)
At risk	62.8 (49)
MST risk category, % (*n*)	
Not at risk	26.9 (21)
Moderate	30.8 (24)
High	42.3 (33)
Primary admission diagnosis (ICD), % (*n*)	
02 Neoplasms (2A00‐2F9Z)	44.9 (35)
12 Diseases of the respiratory system (CA00‐CB7Z)	17.9 (14)
11 Diseases of the circulatory system (BA00‐BE2Z)	9.0 (7)
01 Certain infectious or parasitic diseases (1A00‐1H0Z)	6.4 (5)
13 Diseases of the digestive system (DA00‐DE2Z)	6.4 (5)
08 Diseases of the nervous system (8A00‐8E7Z)	5.1 (4)
21 Symptoms, signs or clinical findings, not elsewhere classified (MA00‐MH2Y)	5.1 (4)
Others	5.1 (4)
Comorbidities, % (*n*)	
Diabetes	57.7 (45)
Hypercholesterolaemia	55.1 (43)
Dementia	51.3 (40)
Chronic obstructive airways disease/asthma	29.5 (23)
Coronary artery disease	29.5 (23)
Depression	25.6 (20)
Osteoarthritis	25.6 (20)
Chronic kidney disease	21.8 (17)
Anaemia	16.7 (13)
Hyperlipidemia	15.4 (12)
Medications, % (*n*)	
Opioids	93.6 (73)
Antiemetics	93.6 (73)
Benzodiazephines	84.6 (66)
Analgesics (paracetamol, NSAIDs)	76.9 (60)
Corticosteriods	70.5 (55)
Antiarrhythmics	69.2 (54)
Antidepressants	69.2 (54)
Antihypertensives	69.2 (54)
Laxatives	55.1 (43)
NMDA‐receptor antagonists	50.0 (39)
Diuretics	46.2 (36)
Somatostatin analogues	37.2 (29)
Antihistamines	35.9 (28)
Antibiotics	34.6 (27)
Anticholinergics	30.8 (24)
Antineoplastic agents	29.5 (23)
Muscle relaxants	26.9 (21)
Bisphosphonates	23.1 (18)
Antiepileptics	19.2 (15)

Abbreviations: ICD, International Classification of Diseases; MST, Malnutrition Screening Tool; RUG‐ADL, Resource Utilisation Group—Activities of Daily Living.

### 6.3 Objective 3

#### 6.3.1 Predictors of Pressure Injury

To determine predictive factors associated with hospital‐acquired pressure injury, a logistic regression model was tested, which contained three scales (AKPS score, PSS score, RUG‐ADL score) and two categorical (gender, deteriorating phase) predictor variables. The predictor variables entered into the model were found to be a good fit (Hosmer and Lemeshow *p* = 0.859) and the full model was statistically significant [*χ* ^2^(5, *n* = 1976) = 16.14, *p* = 0.006]. The model correctly classified 96.1% of cases but explained between only 0.8% (Cox and Snell *R* ^2^) and 2.9% (Nagelkerke *R* ^2^) of the variance in pressure injury incidence. Only one variable made a unique statistically significant contribution: PSS score (Data S3). For each point increase in score, patients were 1.2 times more likely to develop a hospital‐acquired pressure injury (OR 1.17, 95% CI 1.05–1.31; *p* = 0.004).

### 6.4 Objective 4

#### 6.4.1 Characteristics of Pressure Injuries

There were 95 hospital‐acquired pressure injuries across 78 patients. The majority of patients (79.5%, *n* = 62) had only one pressure injury, with most of the remainder having two (19.2%, *n* = 15) and only one patient (1.1%) having three. The median time from admission until pressure injury occurrence was 6 days (IQR 2–12, range 1–35). Most pressure injuries were stage 1 (40.0%, *n* = 38) or stage 2 (19.2%, *n* = 31), with most occurring on the sacrum (30.5%, *n* = 29), heel (12.6%, *n* = 12) or genitals (12.6%, *n* = 12) (see Table 3). Most pressure injuries (85.3%, *n* = 81) occurred when the patient was in the deteriorating phase. However, the most severe pressure injury (stage 3) occurred in a patient during the stable phase. In five cases of pressure injuries (in four patients), the patient's palliative care phase had improved at the time of the pressure injury (see Table 4).

**TABLE 3:** Pressure injury stage by main sites (*n* = 95).

	Stage 1	Stage 2	Stage 3	Unstageable	SDTI	Mucosal	Total
	*n* (%)	*n* (%)	*n* (%)	*n* (%)	*n* (%)	*n* (%)	*n* (%)
Sacrum	10 (34.5) (26.3)	11 (37.9) (35.5)	1 (3.4) 100	4 (13.8) (36.4)	3 (10.3) (25.0)		29 (100) (30.5)
Heel	4 (50.0) (7.9)	2 (16.7) (6.5)		3 (25.0) (27.3)	3 (25.0) (25.0)		12 (100) (12.6)
Genitals	6 (50.0) (15.8)	2 (16.7) (6.5)		1 (8.3) (9.1)	1 (8.3) (8.3)	2 (16.7) (100)	12 (100) (12.6)
Natal cleft	3 (50.0) (7.9)	3 (50.0) (9.7)					6 (100) (6.3)
Coccyx	1 (16.7) (2.6)	5 (83.3) (16.1)					6 (100) (6.3)
Other sites	14 (46.7) (36.8)	8 (26.7) (25.8)		3 (10.0) (27.3)	5 (16.7) (41.7)		30 (100) (31.6)
Total	38 (40.0) (100)	31 (32.6) (100)	1 (1.1) (100)	11 (11.6) (100)	12 (12.6) (100)	2 (2.1) (100)	95 (100) (100)

Abbreviation: SDTI, suspected deep tissue injury.

**TABLE 4:** PC‐phase by pressure injury stage (*n* = 95).

	PC‐phase at time of pressure injury			
	1: Stable (*n*)	2: Unstable (*n*)	3: Deteriorating (*n*)	Total (*n*)
PC‐phase on admission				
1: Stable	1	0	3	4
2: Unstable	0	8	10	18
3: Deteriorating	2	3	68	73
Total	3	11	81	95
Pressure injury stage				
Stage 1	1	4	33	38
Stage 2	1	4	26	31
Stage 3	1	0	0	1
Unstageable	0	1	10	11
SDTI	0	1	11	12
Mucosal	0	1	1	2
Total	3	11	81	95

Abbreviations: PC, palliative care; SDTI, suspected deep tissue injury.

Sixteen (16.8%) pressure injuries were device‐related; associated with either an indwelling urinary catheter (75.0%, *n* = 12) or nasal prongs (25.0%, *n* = 4). Most devices (81.3%, *n* = 13) were inserted in the palliative care unit and three urinary catheters were inserted in other wards prior to admission.

## 7 Discussion

This study is, to our knowledge, the first to specifically examine the cumulative incidence and characteristics of hospital‐acquired pressure injuries in hospitalised adults in the acute phases (stable, unstable, deteriorating) of palliative care.

Between January 2019 and December 2022, a total of 78 hospital‐acquired pressure injury cases were reported, resulting in a cumulative incidence rate of 3.9% among the acute palliative care cohort. Notably, the majority (74.4%) of these patients were in the deteriorating phase when pressure injuries developed. However, no statistically significant difference was observed across the three acute palliative phases (*p* = 0.431) (stable, unstable, deteriorating). In comparison, a period incidence audit (August 2017 to August 2018) conducted at the same palliative care unit (Bradley and Fox 2020 conference abstract) was lower at 2.5%, with the primary focus being patients in the EoL phase. Our reported incidence rate of 3.9% is lower than that of other palliative care studies outside Australia, with Galvin (2002) reporting a pressure injury incidence of 12% in their Palliative Care Unit over 2 years, and a systematic review (Ferris et al. 2019) detailing a pooled‐data range of 10% to 24% and overall incidence of 11.3% across 12 studies. However, studies did not differentiate between acute and EoL palliative patients, making direct comparisons difficult. Thus, the pressure injury incidence for acute palliative care patients in other units remains unclear.

In terms of the assessed level of risk for pressure injury development, in our study, all patients with hospital‐acquired pressure injuries had a Waterlow Score indicating they were at‐risk or higher on admission for pressure injury development. The present finding seems to be consistent with research by Galvin (2002), which found that the Waterlow Score predicted that 95.3% of those in the high‐ and very‐high‐risk groups subsequently developed pressure damage. However, Fromantin et al. (2011) argue that the Waterlow Score is poorly adapted to oncology because it contains irrelevant items, although these are not stated. Walsh and Dempsey (2011) found that the Waterlow Score had poor interrater reliability, while a significant proportion (41%) of their survey respondents criticised the Waterlow Score for having various problems, including that it was non‐specific to palliative care.

Risk assessments (such as the Waterlow Score) provide risk status and level of risk based on specified risk factors (Waterlow 2005), whereas clinical judgement can incorporate other information on the health status of individuals (Webster et al. 2011). Some claim that the Waterlow Score is not superior to clinical judgement for the assessment of pressure injury risk (Webster et al. 2011). On the contrary, Fulbrook et al. (2024) found that nurse assessors using clinical judgement alone could be underestimating risk level; consequently, under‐allocating preventative interventions, which in turn can place patients at risk of pressure injury development. Although there is no contemporary research on how these results impact incidence for acute palliative care, nonetheless, the results from this study highlight that acute palliative patients are a high‐risk cohort for pressure injury development. In the context of acute palliative care, further study focusing on population‐specific risk factors and risk level assessment incorporating clinical judgement is recommended.

Our results indicate that just over half of the patients in our cohort who developed hospital‐acquired pressure injuries were male (51%). The Waterlow Score used at our hospital includes male and female as independent risk factors and suggests that females may be at a higher risk for skin breakdown (Waterlow 2005). Recently, Koban et al. (2024) also found that the risk for pressure injuries increases for females (*p* = 0.08). However, Chung et al. (2022) identified more significant results for male gender as a significant predictor of pressure injury risk; while Lichterfeld‐Kottner et al. (2020) concluded that gender should not be regarded as an independent risk factor. The inconsistent results across studies suggest that gender may not be as influential a factor as the Waterlow Score currently assumes and questions the validity of this score. As such, further research is necessary to determine whether gender should remain a factor in the assessment, and if so, how it should be incorporated into clinical practice.

In this study, the mean age of patients was over 65, with a significant proportion diagnosed with chronic diseases such as cancer and diabetes. Our findings are consistent with the existing literature, which indicates that patients aged 65 and older, particularly those with chronic diseases, are at a higher risk for developing hospital‐acquired pressure injuries (Jaul et al. 2018). Koban et al. (2024) found patients over 65 years of age, especially those with conditions like cancer, diabetes and cardiovascular disease, bear a disproportionate burden of pressure injuries.

Several factors likely contribute to the increased vulnerability of the acute palliative care demographic, including impaired mobility, reduced skin integrity and poor nutritional status. These characteristics are well‐documented as risk factors for pressure injuries (Koban et al. 2024; NPIAP et al. 2019). In our study, malnutrition was identified as a significant risk factor, with 42.3% of the acute palliative care patients in our cohort with hospital‐acquired pressure injuries classified as high‐risk for malnutrition using the MST. This aligns with literature linking poor nutrition and pressure injury incidence (Chen et al. 2023) and malnutrition with a higher risk of pressure injury (Artico et al. 2018; Koban et al. 2024). Therefore, our results reinforce the need for risk assessment and pressure injury prevention to address not only underlying health status but also the associated risk factors, such as malnutrition status and impaired nutrition.

The median length of hospital stay for the acute palliative patients with pressure injury was 14 days, which aligns with findings from Hendrichova et al. (2010), who identified prolonged hospital stays as a statistically significant risk factor for the development of pressure injuries in palliative patients with cancer. Extended hospitalisations can contribute to increased periods of immobility, a key factor in the development of pressure injuries (Hendrichova et al. 2010); a known challenge for palliative care patients (Hendrichova et al. 2010). Given the association that prolonged hospital length of stay increases the risk of pressure injuries, interventions focused on reducing the length of hospital stays, when clinically appropriate, could play a crucial role in preventing the onset of pressure injuries. Shortening hospital stays, while ensuring adequate care and recovery, may not only reduce the burden of pressure injuries but also improve overall patient outcomes by mitigating the risks associated with prolonged immobility.

The anatomical distribution of pressure injuries in our study was in line with the findings of Lyder et al. (2012), where the sacrum (30.5%) was the most common site, followed by the heels (12.6%) and genital areas (12.6%). These areas are particularly susceptible to pressure and shear forces, which are exacerbated by immobility. The sacrum and heels, in particular, are subject to prolonged pressure from lying in bed or sitting for extended periods, making them common sites for pressure injury development. The genital area, often overlooked, is equally vulnerable, as it is exposed to moisture and friction, particularly in individuals who are unable to reposition themselves regularly. These findings underscore the importance of targeted preventive strategies aimed at these high‐risk areas, particularly for patients with compromised mobility or those heavily dependent on caregivers. Regular repositioning, skin assessments, and the use of pressure‐relieving devices are essential interventions in preventing pressure injuries in these vulnerable regions.

When examining the RUG‐ADL assessment criterion for ‘pressure area risk’, patients with a score of ≥ 15 (high care needs) are considered at‐risk. However, our results did not align with the criterion, with only 33 patients (42.3%) classified with a score of ≥ 15, (which is supposed to indicate ‘pressure area risk’). This discrepancy may arise from the different objectives of the two tools: while the Waterlow Score specifically assesses pressure injury risk, the RUG‐ADL primarily evaluates a patient's functional status. This suggests that while functional assessments are valuable, they may not always fully capture the complex factors contributing to pressure injury development, such as immobility, incontinence, or nutritional status, which the Waterlow Score addresses more comprehensively (Waterlow 2005). Furthermore, future investigations into population‐specific risk assessment should incorporate aspects of RUG‐ADL.

Similarly, the AKPS score, which assesses a patient's general well‐being and functional status, did not show a statistically significant association with pressure injury development in our cohort (*p* = 0.096). This is consistent with findings by Artico et al. (2018), who also noted that AKPS was not a strong predictor. However, our study did observe that patients who developed pressure injuries had higher levels of dependence with ADLs and required assistive devices for mobility and bed transfers. This suggests that while AKPS may not be the most sensitive predictor, functional dependency and immobility remain key risk factors.

A noteworthy finding in our study was the association of the PSS score as a predictor for the development of pressure injuries. The PSS is a clinician‐rated score to measure the overall severity of palliative care‐related issues experienced by a patient across four domains—pain, other symptoms, psychological/spiritual and family/carer (Masso et al. 2015). The overall PSS score has been shown to be a robust predictor of pressure injury risk. The regression analysis for PSS was statistically significant (*p* = 0.004) and 1.2 times more likely to develop a pressure injury. These results indicate that a higher PSS score greatly increases the patient's vulnerability to pressure‐related damage. Yet, in the context of pressure injury prevention for acute palliative care, there is no contemporary research to compare our findings. Nonetheless, this highlights the importance of closely monitoring patients with advanced disease, poor mobility and increased symptom severity.

This study provides valuable insights into the incidence and characteristics of hospital‐acquired pressure injuries in acute palliative care settings, underscoring the need for tailored risk assessment and interventions for this high‐risk population. This study is the first to report the cumulative incidence of pressure injuries specifically in acute palliative patients, a gap in the existing literature where previous research typically focused on the EoL phase or included only EoL patients in their palliative care sample. One of the key findings of this study is the association between higher RUG‐ADL and PSS scores in patients who developed pressure injuries. Notably, the PSS score emerged as a significant predictor for pressure injury development, suggesting that higher levels of dependency and symptom severity could increase vulnerability to these injuries. Based on the results from this study, risk assessment may benefit from incorporating RUG‐ADL aspects into existing tools (such as the Waterlow Score) when assessing the risk of pressure injury.

These results provide important evidence that could guide future research for risk assessment specific to acute palliative care patients. Results from this study contribute novel insights to the field, emphasising the relevance of pressure injury prevention across various stages of palliative care. This research expands our understanding of pressure injury incidence in palliative care by addressing a population that has often been overlooked in previous studies and lays the groundwork for future research to explore pressure injury risk assessment tailored to acute palliative patients.

### 7.1 Strengths and Limitations of the Work

Our study offers valuable contributions to the literature on hospital‐acquired pressure injuries in acute palliative care settings. This study is, to our knowledge, the first to specifically examine the cumulative incidence and characteristics of hospital‐acquired pressure injuries in hospitalised adults in the acute phases (stable, unstable, deteriorating) of palliative care. Our secondary analysis of retrospective data suggests that acute palliative care patients may be at a higher risk for developing pressure injuries than thought, as indicated by their Waterlow scores. Furthermore, clinical assessments identified several risk factors for pressure injury development in this cohort, including advanced age, malnutrition and reduced mobility. These findings underscore the need for further research to develop a deeper understanding of pressure injury risk assessment tailored to acute palliative care patients.

One potential limitation of this study is the under‐recognition and under‐reporting of hospital‐acquired pressure injuries. As demonstrated by Graves et al. (2023), such issues are prevalent in clinical settings. For instance, the primary method used by the study hospital to identify hospital‐acquired pressure injury was RiskMan. However, cross‐referencing the QHERS databases with RiskMan revealed 13 hospital‐acquired pressure injury cases that had not been reported in the RiskMan system. This discrepancy highlights the possibility of reporting error, which can be influenced by factors such as time constraints, lack of adequate training and suboptimal documentation practices, which collectively contribute to reporting bias (Carlfjord et al. 2018). This bias may lead to a lower‐than‐actual incidence rate, thus potentially skewing the data.

A key limitation of this study was the inability to conduct further regression analyses, which could have provided a more detailed understanding of the risk factors associated with hospital‐acquired pressure injuries in this cohort. Specifically, the Waterlow Score on admission was not available for acute palliative care patients who did not develop a pressure injury, which would have provided useful comparative data. Additionally, all patients who developed hospital‐acquired pressure injuries were assessed as at‐risk or higher on admission using the Waterlow Score. However, the Waterlow score on the day the pressure injury occurred was not recorded. This gap in data collection limits the ability to fully explore the relationship between baseline risk assessments and the incidence of hospital‐acquired pressure injuries.

While we utilised multiple data sources to track the incidence of hospital‐acquired pressure injuries in the palliative care unit and carefully merged and cleaned these datasets, the lack of comprehensive data for acute palliative care patients without a hospital‐acquired pressure injury represents a notable constraint. This incomplete dataset restricts the potential for more robust regression analyses and deeper insights into patient risk profiles.

### 7.2 Recommendations for Further Research

One of the key challenges identified in this study is the lack of clarity surrounding the definition of palliative care patients in acute hospital settings. This ambiguity makes it difficult to apply and interpret research on pressure injury prevention and management in acute palliative care contexts. To address this, future research should prioritise the development and standardisation of definitions for acute palliative care patients. One promising approach is to adopt the palliative care phases, which have been utilised in Australia for over 20 years (Masso et al. 2015) and are increasingly being explored in other countries such as Germany (Lehmann et al. 2021), Belgium (Leysen et al. 2020) and Taiwan (Hsiao et al. 2022). Defining the acute palliative care cohort more precisely using these phases will enable researchers to better focus on pressure injury‐related outcomes in this specific patient population.

Another important avenue for future research is the need for prospective studies to deepen our understanding of pressure injury risk and the associated factors that contribute to its development within the acute palliative care setting. While retrospective studies have provided useful insights, prospective studies are essential for identifying the unique risk factors specific to this cohort. These studies should focus on relevant variables within Palliative Care Units, considering both patient‐specific and environmental factors that may influence pressure injury development. Such studies could also assess the effectiveness of various preventative interventions and their applicability to acute palliative patients.

In this study, we observed the use of the Waterlow Score in the Palliative Care Unit at the study hospital; however, there is limited evidence supporting the appropriateness of this tool for the acute palliative cohort. Future research should evaluate the effectiveness of existing risk assessment tools, like the Waterlow Score, within acute palliative settings, which was found to be useful in other contexts (Fulbrook et al. 2024).

Future studies can refine nursing care risk assessment and preventative strategies to improve patient outcomes by focusing on evidence‐based risk assessment, incorporating population‐specific aspects (from RUG‐ADL or PSS) in acute palliative care. Moreover, to improve the generalisability of research findings, multi‐site and cross‐cultural studies are strongly recommended. Expanding research beyond single‐site studies will provide a more comprehensive understanding of pressure injury risk and management across diverse healthcare settings. Such studies should involve a variety of Palliative Care Units, both within different regions of Australia and internationally. This would enhance the ability to identify universal trends as well as context‐specific challenges in pressure injury prevention and management, allowing for the development of more broadly applicable recommendations for practice.

Finally, longitudinal studies that track patient outcomes over time will be critical in evaluating the long‐term effectiveness of pressure injury risk assessment in acute palliative care. These studies can provide important data on the validity and reliability of the risk assessment in the context of pressure injury prevention. By addressing these key areas of research, we can improve our understanding of pressure injury risk assessment, prevention and management in acute palliative care, ultimately enhancing the quality of care for this vulnerable patient population.

### 7.3 Implications for Policy and Practice

The findings from this study have important implications for clinical practice, particularly in the risk assessment of pressure injuries in acute palliative care settings. Given that patients in this cohort were at high risk for pressure injury development, strategies for early identification and tailored prevention are critical. Healthcare providers should prioritise regular and comprehensive assessments of pressure injury risk, particularly during the unstable and deteriorating phases of care, when the likelihood of pressure injury formation is heightened.

Validated risk assessment tools such as Waterlow Score are useful but do not capture the full picture of pressure injury risk for this specific cohort. Refinement or adaptation to incorporate RUG‐ADL and AKPS aspects may provide a bespoke tool that better serves the acute palliative patient cohort. Additionally, the use of opioid and antiemetic medications, which can contribute to decreased mobility and increased drowsiness, warrants careful monitoring and proactive management to mitigate the risk of pressure injury development. By integrating targeted pressure injury prevention protocols and individualised care plans, healthcare teams can significantly reduce the incidence of hospital‐acquired pressure injuries, improve patient outcomes and enhance the quality of care in acute palliative settings.

## 8 Conclusion

The incidence rate of hospital‐acquired pressure injuries in acute palliative care patients was found to be lower than the rates reported in the broader palliative care literature. However, these findings are not directly comparable to previous studies conducted with patients at all phases of palliative care, including EoL. Additionally, while acute phases of illness (stable, unstable and deteriorating) were not significantly different in relation to pressure injury development, most hospital‐acquired pressure injuries occurred during the deteriorating phase at admission and persisted in that phase. Based on the results of this single‐site study, we recommend that healthcare providers caring for acute palliative patients consider these factors when implementing comprehensive pressure injury prevention and management strategies.

## Author Contributions

**Saroeun Ven:** conceptualisation, data curation, formal analysis, investigation, methodology, project administration, writing – original draft, writing – review and editing. **Michael Steele:** supervision, conceptualisation, formal analysis, methodology, writing – review and editing. **Adam Burston:** supervision, conceptualisation, methodology, writing – review and editing. **Paul Fulbrook:** supervision, conceptualisation, formal analysis, methodology, writing – review and editing. **Josephine Lovegrove:** supervision, conceptualisation, methodology, writing – review and editing. **Sandra Miles:** supervision, conceptualisation, methodology, writing – review and editing. **Susan Prince:** supervision, writing – review and editing.

## Disclosure

Submission with statistics: There is a statistician on the author team; Dr. Michael STEELE PhD, BSc (Math) (Hons).

## Conflicts of Interest

The authors declare no conflicts of interest.

## Supporting information


Data S1.

Data S2.

Data S3.

## Data Availability

The data that support the findings of this study are available on request from the corresponding author. The data are not publicly available due to privacy or ethical restrictions.
