# Supplementary material for: Incidence and Characteristics of Hospital‐Acquired Pressure Injuries in Acute Palliative Care Patients: A Four‐Year Analysis
Source: J Clin Nurs. 2025 May 19;35(1):255–67. doi: 10.1111/jocn.17829 (PMC12666988; doi:10.1111/jocn.17829)
Supplement: Supplementary file 2 — Data S2. [file JOCN-35-255-s001.docx]

| **Symptom Assessment Scale (SAS)**  Complete Definition | | |
| --- | --- | --- |
| **Patient Rated distress relating to symptoms over a 24hr period** | | |
| The Symptom Assessment Scale describes the patient’s level of distress relating to individual physical symptoms. The symptoms and problems in the scale are the seven most common.  **Usage:**   1. Best practice is for the patient to rate distress either independent or with the assistance of a clinician or family/carer using a visual of the scale such as the *Symptom Assessment Scale Form for Patients*. 2. Symptom distress may be rated by proxy. This only occurs when the patient is unable to participate in conversation relating to symptom distress i.e. Terminal phase. | | **Proxy:** a family / carer or clinician who rates symptom distress on behalf of the patient though observational assessment. Use the following codes to describe Patient = Pt, Fam/Carer= FC or Clinician =Cl  **Instructions**: patient to consider their experience of the individual symptom or problem over the last 24 hours and rate distress according to  A score of **0**: means distress from the symptom absent  A score of **1**: means the symptom is causing minimal distress.  A score of **10**: means the symptom is causing the worst possible distress. |
| **Problem Severity Score (PSS)**  Complete Definition  **Clinician rated assessment of problems over a 24hr period**  Global assessment of four palliative care domains to summarise palliative care needs and plan care.  The severity of problems are rated and responded to following using the scale:  **0 = Absent; 1 = Mild; 2 = Moderate; 3 = Severe**  **Pain:** overall severity of pain problems for the patient  **Other Symptoms:** overall severity of problems relating to one or more symptoms other than pain  **Psychological / Spiritual:** severity of problems relating to the patient’s psychological or spiritual wellbeing. May be one or more issues.  **Family / Carer:** problems associated with a patient’s condition or palliative care needs. Family / Carer do not need to be present to asses needs as written, verbal or observational information may be used. | | **Australia-modified Karnofsky Performance Status (AKPS)**  Complete Definition  **Clinician rated assessment of performance relating to work, activity and self-care over a 24hr period**   1. Normal, no complaints or evidence of disease 2. Able to carry on normal activity, minor signs or symptoms of disease 3. Normal activity with effort, some signs or symptoms of disease 4. Care for self, unable to carry on normal activity or to do active work 5. Occasional assistance but is able to care for most needs 6. Requires considerable assistance and frequent medical care 7. In bed more that 50% of the time 8. Almost completely bedfast 9. Totally bedfast & requiring nursing care by professionals and/or family 10. Comatose or barely rousable |
| **Resource Utilisation Group – Activities of Daily Living (RUG-ADL)**  Abbreviated Definition  **Clinician rated assessment of dependency over 24hr period** | | **Palliative Care Phase**  Abbreviated Definition  **Clinician rated assessment**   1. **Stable** Symptoms and problems are adequately controlled by established management. Monitor, review, anticipate & respond. 2. **Unstable** An urgent change in the plan of care or emergency treatment is required due to development of a new problem &/or a rapid increase in the severity of existing problems &/or family/carer problems. Urgent response required. 3. **Deteriorating** The plan of care is addressing anticipated needs but requires periodic review due to gradual functional decline &/or worsening of existing symptoms &/or the development of new but expected problems &/or family/carer problems. Review & change care plan 4. **Terminal** Death likely in a matter of days. Monitor, review & respond |
| **For Bed Mobility, Toileting & Transfers** | **For Eating** |  |
| 1. Independent or supervision only 2. Limited physical assistance 3. Other than two person physical assist 4. Two or more person physical assist | 1. Independent or supervision only 2. Limited assistance 3. Extensive assistance / total dependence / tube fed |  |
|  | |  |

Supplementary File 2. PCOC Palliative Assessments

Adapted from PCOC https://www.uow.edu.au/australasian-health-outcomes-consortium/pcoc/assessment-tools-and-forms/
