# Supplementary material for: Incidence and Characteristics of Hospital‐Acquired Pressure Injuries in Acute Palliative Care Patients: A Four‐Year Analysis
Source: J Clin Nurs. 2025 May 19;35(1):255–67. doi: 10.1111/jocn.17829 (PMC12666988; doi:10.1111/jocn.17829)
Supplement: Supplementary file 3 — Data S3. [file JOCN-35-255-s003.docx]

Supplementary File 3. Predictor of hospital-acquired pressure injury development.

| Variables | Odds ratio | 95% CI | Wald | *p* Value |
| --- | --- | --- | --- | --- |
| Gender | .687 | 0.426–1.108 | 2.371 | .124 |
| ^1^Deteriorating phase | 1.157 | 0.673–1.989 | .279 | .597 |
| RUG-ADL score | 1.063 | 0.985–1.148 | 2.464 | .116 |
| PSS score | 1.173 | 1.051–1.308 | 8.174 | .004^2^ |
| AKPS score | 1.005 | 0.978–1.033 | .120 | .729 |

Abbreviations: AKPS, Australian-modified Karnofsky Performance Status; CI, confidence interval; RUG-AGL, Resource Utilisation Group – Activities of Daily Living; PSS, Problem Severity Score

^1^Palliative care-phase

^2^ *p*-value of less than 0.05 was considered statistically significant
